# Tumour PD-L1 Expression in Small-Cell Lung Cancer: A Systematic Review and Meta-Analysis

**DOI:** 10.3390/cells9112393

**Published:** 2020-10-31

**Authors:** Emmanuel Acheampong, Afaf Abed, Michael Morici, Samantha Bowyer, Benhur Amanuel, Weitao Lin, Michael Millward, Elin S. Gray

**Affiliations:** 1 School of Medical and Health Sciences, Edith Cowan University, 270 Joondalup Drive, Joondalup, WA 6027, Australia; e.acheampong@ecu.edu.au (E.A.); Afaf.Abed@health.wa.gov.au (A.A.); m.morici@ecu.edu.au (M.M.); BenHur.Amanuel@health.wa.gov.au (B.A.); w.lin@ecu.edu.au (W.L.); 2 Department of Medical Oncology, Sir Charles Gairdner Hospital, Hospital Avenue, Nedlands, WA 6009, Australia; Samantha.Bowyer@health.wa.gov.au (S.B.); michael.millward@uwa.edu.au (M.M.); 3 School of Medicine, University of Western Australia, 35 Stirling Highway, Crawley, WA 6009, Australia; 4 Department of Anatomical Pathology, PathWest, Hospital Avenue, Nedlands, WA 6009, Australia

**Keywords:** programmed death ligand-1, small-cell lung cancer, meta-analysis

## Abstract

Antibodies against programmed death-1 (PD-1), and its ligand, (PD-L1) have been approved recently for the treatment of small-cell lung cancer (SCLC). Although there are previous reports that addressed PD-L1 detection on tumour cells in SCLC, there is no comprehensive meta-analysis on the prevalence of PD-L1 expression in SCLC. We performed a systematic search of the PubMed, Cochrane Library and EMBASE databases to assess reports on the prevalence of PD-L1 expression and the association between PD-L1 expression and overall survival (OS). This meta-analysis included 27 studies enrolling a total of 2792 patients. The pooled estimate of PD-L1 expression was 26.0% (95% CI 17.0–37.0), (22.0% after removing outlying studies). The effect size was significantly heterogeneous (I^2^ = 97.4, 95% CI: 95.5–98.5, *p* < 0.0001).Positive PD-L1 expression was a favourable prognostic factor for SCLC but not statistically significant (HR = 0.86 (95% CI (0.49–1.50), *p* = 0.5880; I^2^ = 88.7%, *p* < 0.0001). Begg’s funnel plots and Egger’s tests indicated no publication bias across included studies (*p* > 0.05). Overall, there is heterogeneity in the prevalence of PD-L1 expression in SCLC tumour cells across studies. This is significantly moderated by factors such as immunohistochemistry (IHC) evaluation cut-off values, and assessment of PD-L1 staining patterns as membranous and/or cytoplasmic. There is the need for large size, prospective and multicentre studies with well-defined protocols and endpoints to advance the clinical value of PD-L1 expression in SCLC.

## 1. Introduction

Lung cancer is the principal cause of cancer-associated mortality globally [1]. Small-cell lung cancer (SCLC) is a devastating subtype of lung cancer that accounts for about 13–15% of all primary cancers of the lung [2]. SCLC has one of the highest mutation rates and is strongly associated with a history of smoking. It is usually diagnosed by bronchoscopic biopsy based on histopathological features and selected neuroendocrine markers [3]. Patients diagnosed with SCLC are staged as an extensive disease (ED) or limited disease (LD) appertaining primarily to the spread of metastatic disease outside the thorax and approximately 70–75% present with an extensive-stage disease at the time of diagnosis [4].

SCLC patients demonstrate better response rates with the current first-line treatments that include platinum-based chemotherapy. However, relapse occurs rapidly in most patients and with the development of acquired drug resistance [5]. The prognosis of SCLC patients continues to be poor with an estimated 5% overall 5-year survival rate [6]. Among the SCLC patients diagnosed with LD, the median overall survival(OS) is 16–24 months with a 2-year survival rate of 25% whilst the median OS among patients with ED is 8–13 months with a 2-year poor survival rate of roughly 5% with standard treatment [5,7,8,9].

Unlike non-squamous non-small-cell lung cancer (NSCLC), SCLC is characterised by a lack of mutually-exclusive, targetable, oncogenic driver mutations [10,11]. Inactivating mutations in the tumour suppressor protein p53 gene (TP53) and retinoblastoma 1 gene (RB1) are the most common recurring mutations in SCLC that cannot be targeted directly [12,13]. Over the last two decades, there has been swift progress in the understanding of the molecular biology of NSCLC and the development of molecular targeted therapy, yet traditional chemo- and radiation therapy for SCLC has remained unchanged [14,15]. Therefore, to improve the treatment outcome of SCLC patients, novel strategies are immediately necessary.

The current successes of cancer-targeted immunotherapies in numerous kinds of cancers have revitalised the hope for better SCLC treatments [15]. Among these greatest successes has been the development of immune checkpoint inhibitors (ICIs), antibodies against programmed death-1 (PD-1), and its ligand, (PD-L1). In cancer tissues, PD-1 is upregulated on tumour-infiltrating lymphocytes (TILs), while PD-L1 is expressed on many types of cancer cells. Cancer cells express PD-L1 to escape immune surveillance via ligation to PD-1 expressed in an adaptive immune response. [16,17,18].

SCLC has been hypothesised to be an immunogenic disease due to the high prevalence of paraneoplastic disorders among SCLC patients [19,20]. Also, the high somatic mutation frequency of SCLC suggests that these tumours are more likely to be immunogenic and could respond to ICIs due to greater variety of neoantigens that can prompt an anti-tumour immune response [21]. Clinical trials have demonstrated that blockade of the interactions between PD-1 and PD-L1 enhances anticancer immunity in SCLC, thus leading to a potentially-improved progression-free survival (PFS) and OS [22,23,24]. This led to the FDA approval of nivolumab, a fully humanised PD-1 ICI antibody, as a third-line treatment for recurrent SCLC in 2018; and of atezolizumab, a fully humanised PD-L1 ICI antibody, as first-line treatment in combination with chemotherapy for extensive diseased staged SCLC (ED-SCLC) in 2019 [25,26]. More recently, durvalumab, a humanised PD-L1 ICI antibody, was also approved in combination with chemotherapy as a first-line treatment for ED-SCLC [27]. The observed clinical benefits in these clinical trials have not been staggering, with a survival benefit of only 2 months and 3 months alongside the addition of ICIs to chemotherapy Hence, biomarkers are needed in the SCLC patient group to help determine who will experience clinical benefit as ICIs have been recognised as a standard treatment option for SCLC.

Several recent studies have determined the expression of PD-L1 protein in SCLC with a range of 0.0–82.8% PD-L1-positive detection rates [28,29,30,31,32,33,34]. Nevertheless, their conclusions are limited due to sample size, antibody clone utilised, staining pattern (membranous and/or cytoplasmic) and cut-off values. In addition, a consistent issue is restricted access to large size and good quality biopsies given that neither repetitive tumour biopsies nor surgical resection are standard of care for SCLC [35]. Additionally, the juxtaposition of the lesions to large blood vessels produces potential complications for trans-thoracic biopsies. These complicating factors have hindered the feasibility of further investigating PD-L1 immunohistochemistry to identify SCLC patients who would benefit from ICIs during treatment [36].

Since anti-PD-1/PD-L1 therapy has been approved for the treatment of SCLC, up-to-date and accurate documentation of PD-L1 expression prevalence is needed to determine if it can serve as a predictive biomarker. Although previous studies have reported on the prevalence of PD-L1 expression in tumour cells for SCLC, there is no comprehensive meta-analysis of the prevalence of PD-L1 expression in SCLC. Here, we performed a meta-analysis on studies conducted to evaluate the prevalence of the expression of PD-L1 on tissue specimens from patients with SCLC and association with OS.

## 2. Materials and Methods

### 2.1. Search Strategy and Selection Criteria

**Figure 1:**
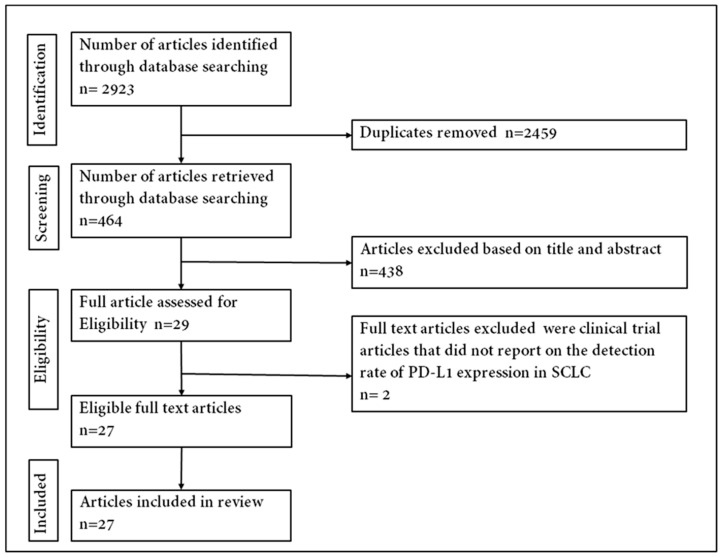
Flowchart of identifying eligible articles.

Using the Preferred Reporting Items for Systematic Reviews and Meta-Analyses (PRISMA) guidelines [37], an online search of literature on PD-L1 expression in SCLC was conducted (Figure 1). The databases searched were the National Center for Biotechnology Information (NCBI) PubMed, Google Scholar, Cochrane Library and EMBASE. All literature searches were performed between 1 November 2019 and 21 May 2020 and were limited to studies conducted in English. The study detailed search parameters that are attached to this manuscript as supplementary figures (Figure S1). Different variations of search text were used in literature with each being an appropriate combination of SCLC-tumour- and PD-L1-associated terms collectively (‘SCLC biopsied tumours’, ‘SCLC resected tumours’, ‘programmed death ligand-1’, ‘programmed cell death ligand-1’, ‘PD-L1’, ‘CD274’ or ‘B7-H1’), disease terms (‘small-cell lung cancer’, ‘SCLC’, ‘small-cell lung malignancy’, ‘small-cell lung neoplasm’ or ‘neuroendocrine carcinoma’) and a combination of terms.

### 2.2. Eligibility and Selection of Articles and Data Extraction

Titles and abstracts of articles were reviewed independently by two authors, strictly using the inclusion criteria stated below. There were yes/no questions for the abstract/title screening process. If all questions were answered yes (or maybe), the article was included for full-text review. The yes/no questions were: (1) Did the study assess the positivity of PD-L1 expression in tumour cells among SCLC patients? (2) Did the study evaluate PD-L1 expression positivity rate and/or clinical outcomes (OS)? (3) Did the study provide a risk analysis of association with clinical outcomes? Articles were excluded upon full-text assessment if they did not meet the criteria. Information extracted from the selected articles included authors’ names, year of publication, sample size, stage of the disease, antibody clones, immunohistochemistry evaluations, PD-L1 positivity rates, cut-off values and prognostic value of PD-L1 expression if it was reported (Table 1). Because this review was to assess the prevalence and/or the prognostic value of positive PD-L1 expression in SCLC, special consideration was given to letter to the editor articles that met the inclusion criteria. The Newcastle–Ottawa Scale (NOS) [38] was used to rate and assess the quality of the full-text articles included in the meta-analysis (Figure S2).

### 2.3. Data Analysis

All data were entered into Microsoft Excel and imported in R software version 3.5.3 for statistical analyses. To minimise the effects of studies with extremely high or low prevalence estimates on the overall pooled estimate, the Freeman–Tukey double arcsine transformation (PFT) was used before pooling. A 95% confidence interval was used in assessing the individual study proportion and pooled effects. The pooled estimates of the prevalence of PD-L1 expression were calculated by the random-effect model. Evaluation of heterogeneity between studies was conducted using Cochran’s heterogeneity statistics (Q) (chi-squared test; χ^2^) and the degree of inconsistency (I^2^).

Heterogeneity (I^2^) in the measure of association across studies was further quantified with the I^2^ statistic, with a value of <25% indicating low heterogeneity, 25–50% indicating moderate heterogeneity, 50–75% indicating high heterogeneity and >75% indicating extreme heterogeneity [39]. The robustness of the pooled effects and possible outliers were evaluated with the leave-one-out sensitivity analysis. A subgroup analysis was performed to explore the possible heterogeneity among studies. The publication bias was assessed using Begg’s funnel plots and confirmed by Egger’s test [40].

## 3. Results

A total of 2923 published articles was identified through the database search. Titles and abstracts/summaries of these articles were screened for significance, and 2894 were excluded by the condition of duplication, reviews, comments, incomplete data and case studies leaving 29 articles to examine for eligibility. Two clinical trial studies that did not report on the detection rate of PD-L1 expression were excluded [41,42]. Paz-Ares et al. [43] and Reck et al. [44], included in this meta-analysis, were further reports from clinical trials that did not initially report on PD-L1 expression. The primary publications of these trials were excluded from the review and meta-analysis. A total of twenty-seven published studies were included in the analysis to address the prevalence and/or prognostic value of positive expression of PD-L1 in SCLC tumours. The steps used in acquiring the published articles included in this review are depicted in Supplement Figure S3 following the PRISMA chart.

Table 1 shows a summary of the publications and details included in the metanalysis. All studies were published between 2015 and 2019, with fifteen of them published between 2018 and 2020. The sample size of the included studies ranged from 30 to 277 SCLC patients with a combined sample size of 2792 SCLC patients. All studies employed immunohistochemistry for measuring and evaluating PD-L1 expression except Carvajal-Hausdorf et al. who utilised multiplexed quantitative immunofluorescence (QIF) for PD-L1 expression measurement and assessment [45]. The most common PD-L1 antibodies used across the studies were clone 22C3 (n = 6) and clone E1L3N (n = 6) followed by clone SP142 (n = 3), clone 28.8 (n = 3), clone 2B11D11 (n = 3), clone EPR1161 (n = 2), clone SP263 (n = 2), clone 5H1 (n = 1) and MAB1561 (n = 1), respectively (Table 1). The studies were conducted in various region of the world with the majority of the studies (n = 15) coming from Asia, specifically, China (n = 7), followed by Japan (n = 6), United States (n = 5), Italy (n = 2), multi-nationals (n = 3) and one from each of the following countries—Germany, Italy, South Korea and Taiwan.

Twenty studies retrospectively assessed the expression of PD-L1 in SCLC tumour cells while seven studies were clinical trials that reported the prevalence of PD-L1 expression in tumours. More than half of the studies (n = 12) used both limited- and extensive-staged SCLC patients, eleven had only extensive-staged patients while two studies constituted only limited-stage SCLC patients. Twelve studies correlated PD-L1 expression with clinical outcomes including OS. Ten of these studies provided statistical analysis on the high-risk association (hazard ratio; HR) with OS, with two demonstrating statistically-significant difference in median OS (mOS) probabilities [28,34].

### 3.1. Prevalence of PD-L1 Expression in SCLC

**Figure 2:**
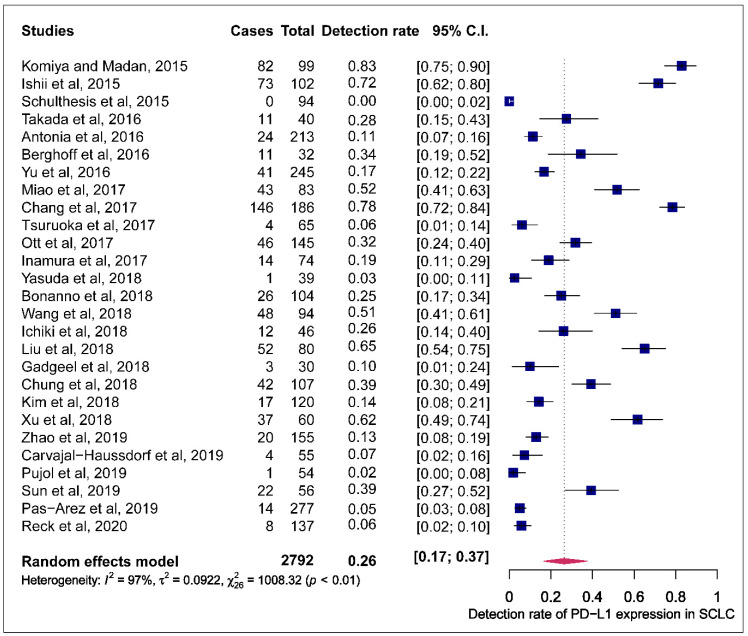
Forest plot of studies reporting the detection rate of programmed cell death ligand-1 (PD-L1) expression in small-cell lung cancer (SCLC). The PD-L1 detection rates and 95% CI of each study are represented with a horizontal line and the square area mirrors the point estimate of each study. A random-effect model was utilised.

The prevalence of PD-L1 expression in SCLC was retrieved from all 27 publications included. PD-L1 positivity was defined by tumour proportional score (TPS) and combined proportion score (CPS). Three of the 27 articles used CPS while the rest employed TPS to define positive PD-L1 expression. One study that assessed positive PD-L1 expression by comparing different antibodies, to minimise the effect of this study on the pooled estimates of PD-L1 expression prevalence, the detection rate recorded by the 28.8 PD-L1 antibody assay was used in the metanalysis [29]. The reported prevalence of PD-L1 expression in these studies ranged from 0.0 to 82.8%. The pooled estimate of the prevalence of PD-L1 expression in SCLC was 26.0% (95% CI: 16.9.0–37.5). The observed effect size was significantly heterogeneous (I^2^ = 97.4, 95% CI: 95.5–98.5, *p* < 0.0001) (Figure 2).

**Figure 3:**
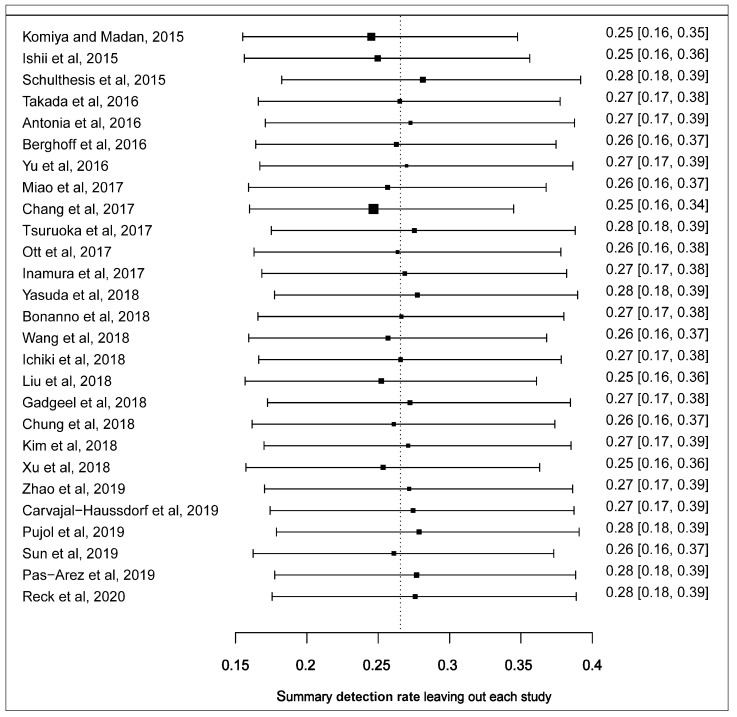
Leave-one-out sensitivity plot of studies reporting the prevalence of PD-L1 expression in SCLC. Each box depicts a summary of the calculated prevalence leaving out a study. The reference shows where the original summarised prevalence lies.

We performed the leave-one-out sensitivity analysis to identify the outlying studies that had a potential influence on the effect size of the forest plot. Four studies including Komiya and Madan, 2015; Schultheis et al., 2015, Ishii et al., 2015 and Chang et al., 2017 [31,32,34,46] had a more pronounced impact on the pooled estimated prevalence of PD-L1 expression. Represented boxes deviated further from the reference line (Figure 3). After the potential outlying studies were left out of the random-effect model, the pooled estimate of the prevalence of PD-L1 expression in SCLC was 22.0% (95% CI: 15.0–30.0) with a significant heterogeneity (I^2^ = 95.0, 95% CI: 91.6–97.5, *p* < 0.0001) (Figure S4).

**Figure 4:**
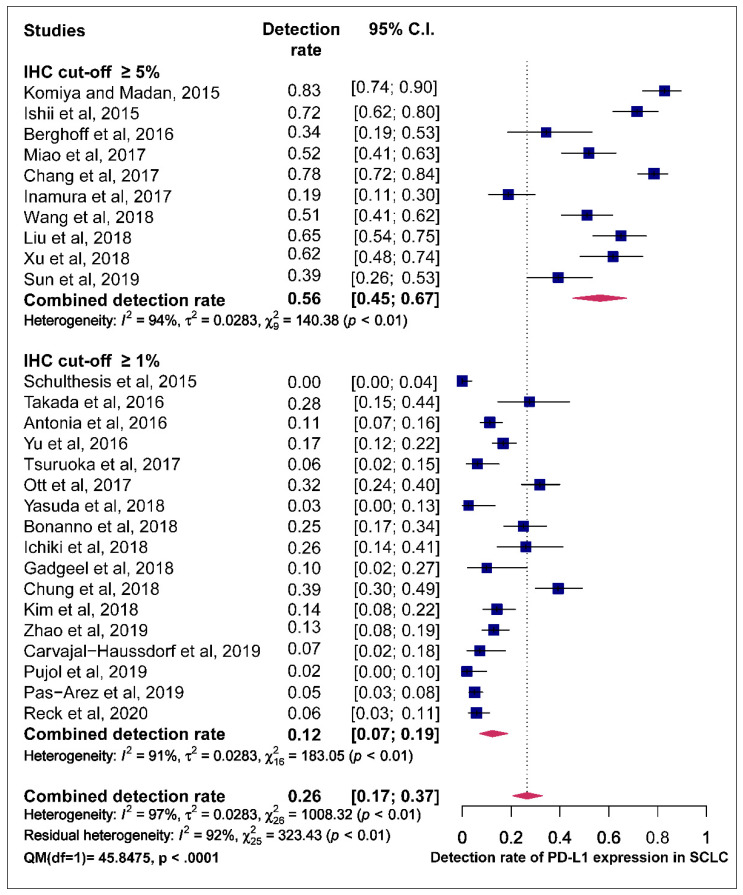
Forest plot of subgroup analysis of the association between immunohistochemistry (IHC) cut-off values and prevalence of PD-L1 expression. The PD-L1 detection rates and 95% CI of each study are represented with a horizontal line and the square area mirrors the effect size of each study A random-effect model was utilised.

The funnel plot was performed to determine publication bias across studies for the prevalence of PD-L1 expression. There is an uneven distribution of points and the plot is asymmetrical. The funnel plot did not reveal any publication bias following the Egger’s test (*p* = 0.6805) (Figure S3). Articles included in the meta-analysis were stratified by positive immunohistochemistry (IHC) PD-L1 expression cut-off values of ≥5% and ≥1% membranous or membranous and cytoplasmic staining of the tumour cells. Studies that employed a cut-off of ≥5% recorded a higher pooled estimate of the prevalence of PD-L1 expression (56.0%, 95% CI (45.0–67.0%)) with significant heterogeneity (I^2^ = 94.0% *p* < 0.01) than those that used a cut-off value of ≥1% (12.0%, 95% CI (7.0–19.0%); I^2^ = 91.0% *p* < 0.01). A statistically-significant difference between ≥5% and ≥1% IHC cut-off values for pooled estimates was observed based on the test of moderators (QM) (QM (1) = 45.8, *p* < 0.0001) (Figure 4).

**Figure 5:**
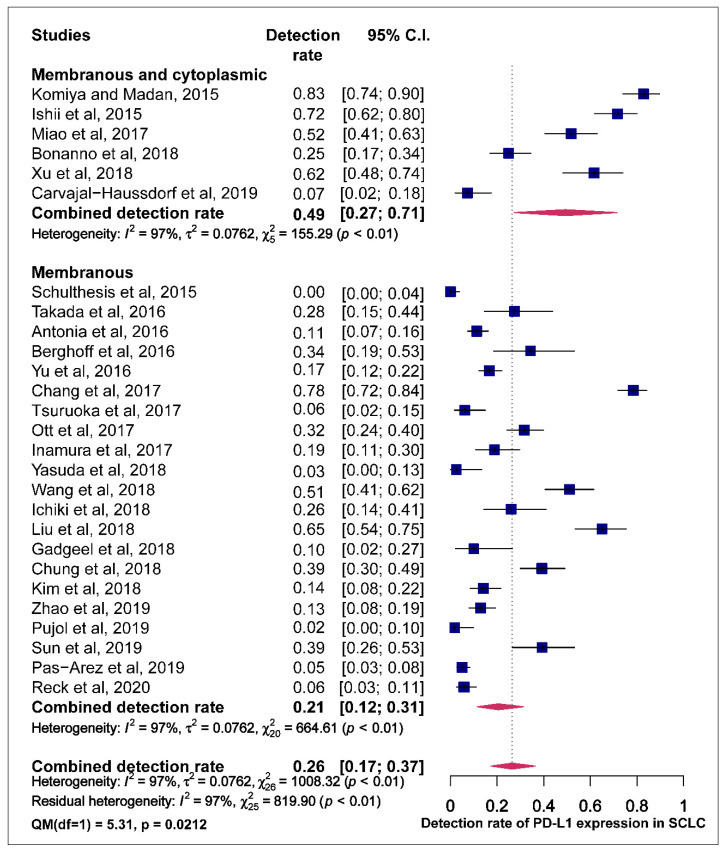
Forest plot of subgroup analysis of the association between the assessment of PD-L1 staining pattern in membrane+/-cytoplasm and prevalence of PD-L1 expression. The PD-L1 detection rates and 95% CI of each study are represented with a horizontal line and the square area mirrors the size effect of each study.

Studies were classified based on the assessment of the PD-L1 staining pattern. Studies that observed staining for PD-L1 in both membrane and cytoplasm recoded higher pooled estimates of PD-L1 prevalence compared to those who observed PD-L1 staining only in the membrane with a statistically significant difference (49.0% vs. 21.0% QM = 5.308, *p* = 0.0212) (Figure 5).

**Figure 6:**
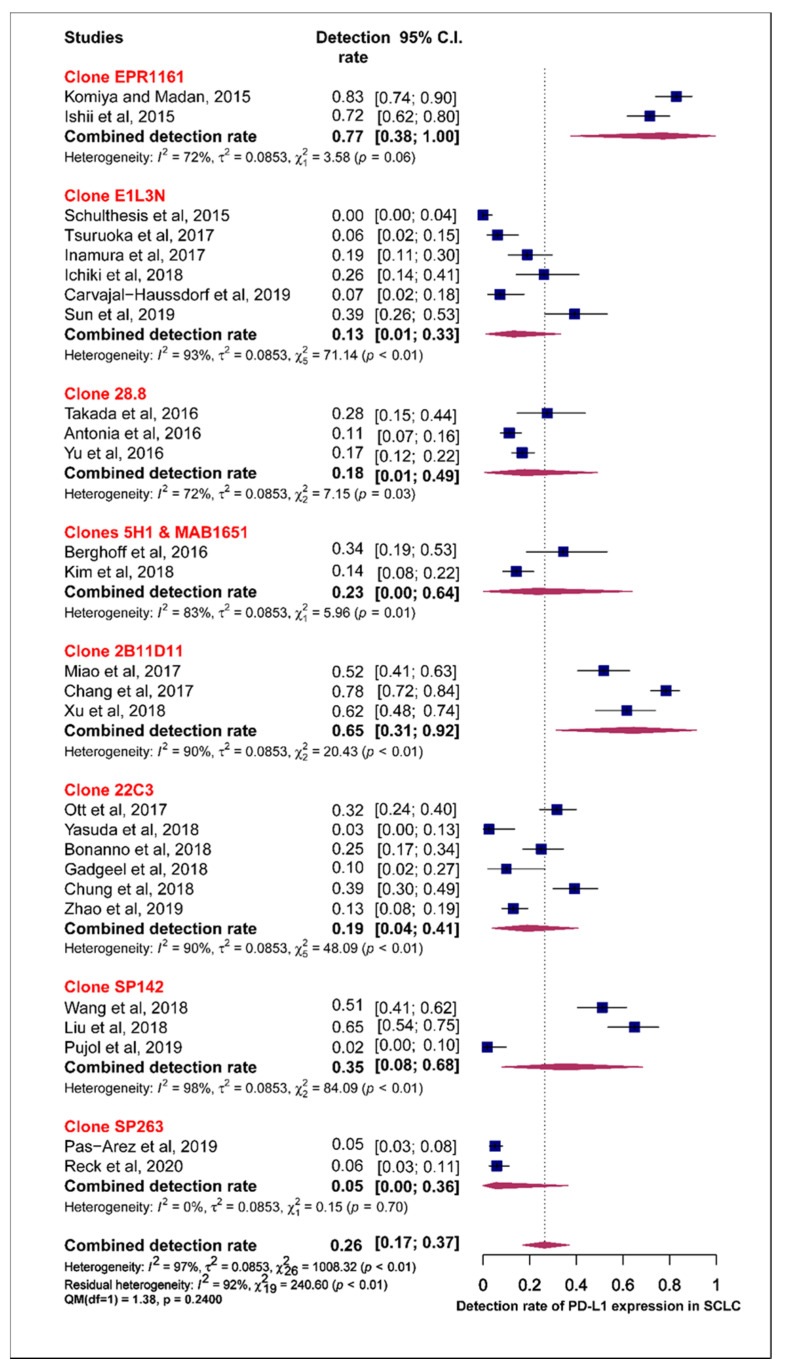
Forest plot of subgroup analysis of the association between the type of antibody and prevalence of PD-L1 expression. The PD-L1 detection rates and 95% CI of each study are represented with a horizontal line and the square area mirrors the size effect of each study. Highlighted in red are the antibody clones from FDA approved PD-L1 assays.

Subgroup analysis was also performed for the individual PD-L1 antibody assays used across the different studies. The pooled estimates for the prevalence of tumour PD-L1 expression for clone 28.8, 22C3, SP142 and SP263 were 18.0%, 19.0%, 35.0% and 5.0%, respectively. The test of moderators did not reveal any statistically significant difference in the pooled estimates (QM (1) = 0.17, *p* = 0.6798) (Figure 6).

Further subgroup analysis reveals that used FDA approved PD-L1 antibody had lower pooled estimates compared to those that did not use (19.0.0%, vs. 35.0). However, there was no significant difference in the pooled estimate on the prevalence of PD-L1 expression (QM (1) = 2.69, *p* = 0.1008) (Figure S5).

Articles were classified based on geographical regions (Asia and others). Articles carried out in Asia observed a high pooled estimate compared to articles from other regions irrespective of the cut-off values for PD-L1 staining with a significant difference between the two groups for PD-L1 prevalence (36.0% vs. 16.0%, QM = 4.46, *p* = 0.0347) (Figure S6).

A meta-regression analysis demonstrated that a unit increases in the study’s sample size affects a 0.2% decrease in the detection rate of PD-L1 expression. However, sample size was not a statistically-significant moderator of the pooled estimated prevalence of PD-L1 expression (QM (1) = 0.27, *p* = 0.6008), which was evident by the insignificant regression constant (R^2^ = −0.002, *p* = 0.6008). However, it was noted that studies with a smaller sample size had low detection rates (Figure S7).

Another meta-regression plot significantly shows that the poor-quality studies tend to have higher Fisher’s Z scores of the effect sizes. In contrast, the better quality studies tend to have lower scores (QM (1) =6.99, *p* = 0.0082, R^2^ = 0.059, *p* = 0.0082), indicating that quality of a study is an important moderator of the pooled estimate of the prevalence of PD-L1 expression in SCLC (Figure S8).

### 3.2. Effect of PD-L1 Expression in Survival

Overall, ten clinical studies stated HR values and 95% confidence intervals (CIs). Nine of these reported HR values and CIs to evaluate positive PD-L1 expression with OS. Only Chang et al. reported HR value and CI for negative PD-L1 expression. Individually, six studies showed PD-L1 to be associated with better overall OS with two studies recording statistical significance. Three studies demonstrated a statistically significant association of PD-L1 expression with shorter OS.

**Figure 7:**
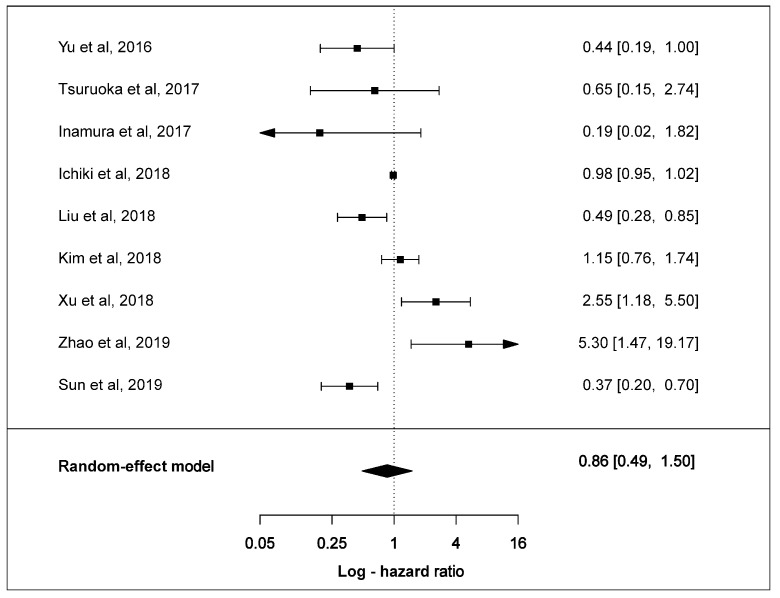
Forest plots of overall survival and PD-L1 expression in SCLC tumours. The HR and 95% CI of each study is represented with a horizontal line and the square area mirrors the size effect of each study. Pooled HR and 95% CI are depicted by diamonds. A random-effect model was utilised.

The estimated pooled HR for nine studies was calculated utilising a random-effect model because the heterogeneity across studies was statistically-significant (χ^2^ =34.3, *p* < 0.001, I^2^ = 88.7%). The pooled HR of all studies was 0.86 (95% CI: 0.49–1.50, *p* = 0.5880) indicating that positive PD-L1 expression showed a trend towards longer OS in SCLC patients (Figure 7). The Egger test demonstrated that Begg’s funnel plot was insignificantly asymmetrical (*p* = 0.7944) (Figure S9).

## 4. Discussion

The potential of applying ICIs for the treatment of SCLC became apparent after the promising results observed in non-small-cell lung cancer, melanoma and other cancer subtypes [61]. High tumour mutational burden has been associated with response to ICIs in several tumours including NSCLC [62,63]. SCLC is a carcinogen-related cancer, with a high frequency of mutation per megabase (7.37 mut/Mb) [11,63,64]. It is, therefore postulated, that SCLC islikely to respond to ICIs because a high variety of neoantigens can prompt an immune-mediated response [62]. This instigated the pursuit of anti-PD1/PD-L1 treatments for SCLC, independent of PD-L1 expression.

The present study provides a systematic review and meta-analysis of PD-L1 expression in SCLC. The pooled prevalence of the expression of PD-L1 in SCLC tumours is 26.0%, and 22.0% after removal of potential outlying studies. However, there were large differences in the rate of PD-L1 expression in SCLC tumours between the studies included, varying from 0% to 82.8%. For instance, Ishii et al. reported a high PD-L1 expression rate while Schultheis et al. showed that PD-L1 expression was absent in SCLC tumour cells (71.6% vs. 0.0%). This discrepancy is due to different clones of antibodies and/or scoring systems. Additionally, Ishii et al. restricted their study population to SCLC patients whose tissue specimens were obtained principally from primary tumours (81.4%), thus presumably most specimens were collected through biopsies. Conversely, Schultheis et al. show that majority of the specimens were derived via resection (54.0%) [32,34]. With the exclusive use of resected specimens, it is possibly biased as only a small percentage of SCLC tumours get resections, notwithstanding that they are in a better prognosis group. On the other hand, the exclusive use of resected samples may minimise sampling error if PD-L1expression on both tumour and immune cells is considered in the scoring algorithm. Overall, the observed pooled prevalence of PD-L1 expression in tumours is lower compared to what has been reported for non-small-cell lung cancer (NSCLC) [47,65,66,67].

Due to the presence of substantial heterogeneity, further analyses were warranted to identify potential factors that can explain the inconsistencies between pooled estimates across studies. Factors such as whether an FDA-approved PD-L1 antibody was used or not did not provide a significant effect on the overall pooled estimated PD-L1 prevalence in SCLC in these studies. Also, meta-regression analysis did not show any significant association between sample size and the pooled estimated prevalence of PD-L1 expression.

However, the application of sub-analysis indicated that IHC evaluation cut-off values (≥1% and ≥5%) had a significant moderating effect on the pooled prevalence estimates, and that could underscore the true heterogeneity in the pooled estimates of PD-L1 prevalence in this meta-analysis. Studies that employed a cut-off of ≥5% recorded a higher pooled estimate of the prevalence of PD-L1 expression compared to those that used a ≥1% cut-off for PD-L1 IHC evaluation. This association between high cut-off score and high detection rates, seems, at first sight, counterintuitive, but it might be the result of the investigators adapting their cut-off given the intensity and prevalence of PD-L1-expressing cells in their analysis. For instance, Tsuruoka et al. and Inamura et al., employed the same antibody for PD-L1 staining and used different cut-off values for PD-L1 positivity with Inamura et al. having a higher detection rate [48,49].

Currently, there are separate scoring systems for PD-L1 staining in NSCLC tumour cells with four FDA-approved PD-L1 assays (antibodies: 22C3, 28.8, SP263 and SP142) on two different platforms (Dako, Ventana) [68,69,70,71,72]. Twelve out of the 25 studies included in this review exclusively used commercially-available FDA-approved PD-L1 antibodies. Only Takada et al. used three different PD-L1 antibodies (28.8, SP142 and E1L3N), of which two are FDA-approved, in a study that immunohistochemically analysed PD-L1 expression in surgically-resected SCLC [28,29]. The remaining 12 studies did not use FDA-approved PD-L1 antibodies; two of these studies reported a high expression of PD-L1 using antibodies utilised that been discontinued due to the lack of specificity for PD-L1 [31,34]. We observed that studies that did not use FDA-approved PD-L1 antibodies recorded high prevalence for PD-L1 expression, compared with those that utilised the FDA-approved PD-L1 antibodies. However, this difference was not statistically significant. Nevertheless, the detection rates of PD-L1 expression were coherent among studies employing FDA-approved assays with the same staining pattern and cut-off values [31,34].

There have been multiple reports indicating that the positivity of PD-L1 definitions is not the same for the different approved and commercially-available PD-L1 assays [73,74,75,76]. In three of the FDA-approved PD-L1 assays, 28.8, SP263 and 22C3, the positivity of PD-L1 staining is defined as complete-circumferential or partial-linear plasma membrane staining of tumour cells at any intensity. Most antibodies to PD-L1 in use are directed to its extracellular domain and immunohistochemically stain tumour tissue with a mixture of cytoplasmic and membrane staining. Cytoplasmic staining obscures the interpretation of a positive reaction on the tumour cell membrane, and thus affects the accuracy of PD-L1 scoring. For scoring purposes, cytoplasmic staining in tumour cells is not considered positive [73,74,75]. On the other hand, PD-L1-positive immune cells, as well as the tumour cells, are considered in the criteria of positive PD-L1 staining in the use of SP142 antibody clone [76]. Unsurprisingly, in our findings, the pooled estimates for PD-L1 expression among studies that use SP142 PD-L1antibody assay were higher than pooled estimates from those that used 22C3 and 28.8 PD-L1 antibody assays. Additionally, the results of the test for heterogeneity in our meta-analysis indicated that the PD-L1 staining pattern criteria, membranous alone vs. membranous and cytoplasmic, significantly explained the variation in the pooled estimate of the prevalence for PD-L1 expression. To some extent, this hinders the attempt to establish one PD-L1 IHC test and contributes to the inconsistencies across studies.

A comparative study, (Blueprint phase I and II projects) to assess the feasibility of harmonising the clinical use of these independently-developed commercially- and FDA-approved PD-L1 IHC assays for PD-L1 detection has been conducted for NSCLC. Both phases I and II of the Blueprint project demonstrated that three (28.8, 22C3 and SP263) of the four assays can be used interchangeably for NSCLC tumour staining whereas the fourth (SP142) constantly stained fewer tumour cells [71,72]. Notably, results from the phase I of the Blueprint project revealed that the detection rates of PD-L1 expression were 60.5%, 60.5% 78.9% and 52.6% for clones 28.8 (1%TPS), 22C3 (1% TPS), SP142 (TC1+/-IC1) and SP263 (25%TPS), respectively [71,72]. These findings are congruent with our meta-analysis results taking into consideration that the expression of PD-L1 has been reported to be proportionally low in SCLC in most studies compared to PD-L1 expression in NSCLC [25,74,77,78]. Specifically, the low detection rates observed for both clones 28.8 and 22C3 (60.5%) in comparison with a high detection rate for SP142 (78.9%) in the Blueprint project, are consistent with the pooled estimates recorded for clones 28.8 (18.0%), 22C3 (19.0%) and SP142 (35.0%) in our meta-analysis results.

Unlike in NSCLC, there has been no large scale ‘harmonisation’ study to examine the performance of different PD-1 IHC tests on the same specimens of SCLC patients. There is a need for studies such as the Blueprint projects for evaluation of PD-L1 expression in SCLC tumours among a large cohort of patients. As a first step, Takada et al. in their study conducted detailed PD-L1 expression analyses in surgically-resected specimens utilising different antibodies and positive cut-off values. The authors also carried out an exhaustive evaluation not only for tumour cells but also for immune and tumour cells together [29].

High expression of PD-L1 has been observed in several solid tumours and previous studies have demonstrated a statistically significant association of PD-L1 expression with response to PD-1/PD-L1 therapies in previously-treated patients with advanced NSCLC [79,80,81]. Moreover, several studies have concluded that high expression of PD-L1 in tumours was associated with shorter survival in meta-analyses of PD-L1 expression in NSCLC [81,82]. On the other hand, our meta-analysis indicates that positive expression of PD-L1 appears to confer longer OS of SCLC patients. While this correlation was not statistically significant, it was consistent with previous reports [47,83]. Zhang et al. 2017 reported that PD-L1 expression was a poor prognostic indicator for NSCLC and pulmonary lymphoepithelioma-like carcinoma (LELC) but not for SCLC [83]. Moreover, the observed longer survival benefits could also be due to the fact that four out of the nine studies that assess the association between PD-L1 expression and OS in this meta-analysis recruited limited-stage SCLC patients while the rest enrolled both limited- and extensive-staged SCLC patients. The prevalence of PD-L1 expression in patients with NSCLC ranges from 50% to 70%, however, the expression of PD-L1 has been reported to be proportionally low in patients with SCLC with most studies demonstrating less than 50% PD-L1 expression. Previous studies have reported that PD-L1 expression in SCLC tends to be lower in advanced disease stages compared to earlier disease stages [50]. For the most part, it is evident in the literature that positive expression of PD-L1 occurs more frequently on tumour-infiltrating immune cells within the SCLC, compared to PD-L1 expression on tumour cells, and high PD-L1 expression on the infiltrating immune cells has been associated with favourable clinical outcome in SCLC patients [28,51,52,53].

There are several limitations to the current study that should be noted. Most of the studies included in the review and meta-analysis are retrospective in nature and have relatively-small sample sizes. Analytical factors such as the type of specimen used for analysing PD-L1 expression (biopsy specimen vs. excision specimen), the use of different assays to assess PD-L1, varied cut-off values, different scoring algorithms (tumour proportional scores vs. combined proportion scores) and the pattern of staining (membranous vs. cytoplasmic) for PD-L1 positivity assessment, significantly varied between studies. Finally, outcome readouts can be influenced by the lack of standardisation of treatment regimens, which may affect survival. Although we perform subgroup analysis and meta-regression, it quite possible that unidentified factors contributed to the significant heterogeneity persisting in the results. The real heterogeneity can be ascribed to methodological and/or clinical variation, specifically systemic diversities between studies beyond what would be expected by chance, such as sample characteristics, study settings, study designs and interventions, and any combination of such factors. Therefore, data results should be generalised and interpreted with caution.

Ultimately, the main goal is to evaluate whether PD-L1 expression as a predictive biomarker to select SCLC patients that will benefit from immunotherapy. However, tumour tissue is needed to carry out this test. Fine-needle aspiration only provides a limited amount of tumour sample for diagnostic analysis, which commonly is not adequate for molecular testing or to accurately assess PD-L1 expression by IHC and has high background stromal cells [84,85]. Moreover, the mutational status of tumours may be altered during therapy which necessitates the need for consecutive samplings. But then again, re-biopsy after initial therapy is not always possible in SCLC patients [86,87].

It is anticipated that circulating tumour cells (CTCs) can serve as a non-intrusive, episodically, and real-time substitute for tumour biopsies for assessing PD-L1 expression in SCLC in the future. SCLC is distinguished by having a larger number of CTCs in extended and recurring diseases compared to other carcinomas [88,89]. Recent studies have demonstrated how the expression of PD-L1 in CTCs could be employed to identify patients with NSCLC for anti-PD-1/PD-L1 therapy [90,91,92,93]. Moreover, the expression of PD-L1 evaluated using CTCs could represent the sum of metastatic sites within a patient, which might overcome the heterogeneity of PD-L1 expression.

## 5. Conclusions

The evidence from this study suggests that there are differences in the prevalence of PD-L1 expression in SCLC tumour cells across studies. The pooled prevalence of PD-L1 expression is lower compared to what is reported in NSCLC and is significantly influenced by IHC evaluation cut-off values, assessment of PD-L1 staining pattern and the quality of the study’s methodological characteristics. Although positive PD-L1 expression in SCLC appears to confer better OS in SCLC patients, its use as prognostic index warrants further studies due to significant variations. Given the prospect of PD-L1 evaluation to impact clinical outcomes of SCLC patients treated with ICIs, there is a need for large, longitudinal, multicentre studies with well-defined protocols and endpoints to advance the clinical value of PD-L1 expression.

## Figures and Tables

**Table 1:** Prevalence of programmed death ligand-1 (PD-L1) on tumour cells in SCLC.

References (Sample Size)	Samples	Stage of Disease	Antibody	IHC/QIF Evaluation (%)	PD-L1 Positivity Rate (%)	Prognosis (Cut-off Value, mOS, HR and *p*-Value)
Ott et al. 2017 [23] (n = 145)	Biopsy tissue (n = 132) Fresh tissue (n = 12)	Extensive	Clone 22C3 antibody (Merck & Co, Kenilworth, NJ, USA)	Membranous ≥ 1%	31.7% (46/145)	n/a
Antonia et al., 2016 [25] (n = 213)	Biopsy tissue	Extensive	Clone 28-8; Epitomics Inc, Burlingame, CA, USA)	Membranous ≥ 1%	11.3% (24/213)	n/a
Yu et al., 2016 [28] (n = 249)	Biopsy tissue	Extensive (n = 96) Limited (n = 98)	Clone SP142 Dako clone 28.8 pharmDx kit	Membranous ≥ 1%	16.5 (41/245)	≥1% PD-L1/TPS, mOS: 9.87 vs. 16.13 months, Log rank test *p* = 0.0511 HR = 0.44 (95% CI 0.18–1.07, *p* = 0.055
Takada et al., 2016 [29] (n = 40)	Surgically-resected tissue	n/a	Clone E1L3N (Cell Signalling Technology, Cambridge, UK),	E1LN–membranous Allred, ≥1%, ≥5%	22.5%, 27.5% 35.0%	N/E
			Clone 28.8 (Abcam Cambridge, UK) Clone SP142, Spring Bioscience, Ventana Tuscon, AZ, USA)	28.8-membranous Allred, ≥1%, ≥5% SP142-membranous Allred, ≥1%, ≥5%	20.5%, 27.0%, 32.5% 15.%, 15.0%, 15.0%	N/E
Miao et al., 2017 [30] (n = 83)	Biopsy tissue	Extensive (n = 36) Limited (n = 47)	Clone 2B11D11/PD-L1/CD274 antibody, Clone SP66, SPRINGBIO, USA)	Membranous and/or cytoplasmic ≥ 5%	51.8% (43/83)	≥5% PD-L1/TPS (mOS, 17.0 vs. 9.0, Log rank test *p* = 0.018) HR not provided
Komiya and Madan, 2015 [31] (n = 99)	Unknown	n/a	EPR1161 (Abcam, Cambridge, UK)	Membranous and/or cytoplasmic ≥ 5%	82.8% (82/99)	NE
Schultheis et al., 2015 [32] (n = 94)	Surgically-resected (n = 51), Biopsy (n = 43)	Extensive (n = 49), Limited (n = 49)	Clone 5H1 (Lieping Chen Laboratory, Yale University, New Haven, CT, USA) Clone E1L3N (Cell Signalling Technology, Cambridge, UK)	Membranous ≥ 1%,	0.0% (0/94)	NE
Yasuda et al., 2018 [33] (n = 39)	Biopsy tissue	Extensive	Clone 22C3 pharmDX (Agilent Technologies, Santa Clara, CA, USA) 22C3 (Dako, Carptintera, CA, USA)	Membranous ≥ 1%	2.5% (1/39)	N/E
Ishii et al., 2015 [34] (n = 102)	Biopsy tissue	Extensive (n = 61), Limited (n = 41)	EPR1161 (Abcam, Cambridge, UK)	Membranous and/or cell-surface ≥ 5%	71.6% (73/102)	≥5% PD-L1/TPS mOS: 16.3 vs. 7.3 months Log-rank test *p* < 0.001 HR not provided
Pas-Ares et al., 2019 [43] (n = 277)	Biopsy tissue	Extensive	Clone SP263 Ventana PD-L1 Assay	Membranous ≥ 1%	5.1 (14/277)	n/a
Reck et al., 2020 [44] (n = 137)	Biopsy tissue	Extensive	Clone SP263 Ventana PD-L1 Assay	Membranous ≥ 1%	5.8 (8/137)	n/a
Carvajal-Hausdorf et al., 2019 [45] (n = 55)	n/a	Limited (n = 23) Extensive (n = 32)	Clone E1L3N (Cell Signalling Technology, Cambridge, UK),	Membranous and/or cytoplasmic ≥ 1%	7.3% (4/55)	NE
Chang et al., 2017 [46] (n = 186)	Biopsy tissue	Limited (n = 74) Extensive (112)	Clone 2B11D11, PD-L1/CD274 antibody (Cat/no.66248-1-Ig) Proteintech Group Inc Chicago, IL, USA	Membranous ≥ 5%	78.0% (145/186)	HR = 0.17 (95% CI: 0.08–0.35, *p* < 0.001)
Sun et al., 2019 [47] (n = 56)	Surgically-resected/biopsy tissue	Limited (n = 25) Extensive (n = 31)	Clone E1L3N, diluted 1:100; #13684, Cell Signalling Technology)	Membranous ≥ 5%	39.3% (22/56)	Overall survival (OS) ≥ 5% HR = 0.37(95% CI: 0.21–0.68), *p* = 0.002
Inamura et al., 2017 [48] (n = 74)	Surgically-resected tissue	Extensive	Clone E1L3N (Cell Signalling Technology, Cambridge, UK),	Membranous ≥ 5%	18.9% (14/74)	Lung-cancer-specific survival months, HR = 0.11 (95% CI: 0.006–0.52, *p* = 0.0020) Overall Survival (OS): HR = 0.19(95% CI: 0.10–1.30, *p* = 0.150)
Tsuruoka et al., 2017 [49] (n = 65)	Biopsy/cytology	Limited	Clone E1L3N (Cell Signalling Technology, Cambridge, UK),	Membranous ≥ 1%	5.8% (4/65)	≥1% PD-L1/TPS, mOS (38 vs. 140 months) Log rank test *p* = 0.067) HR= 0.65(95% CI: 0.16–2.71, *p* = 0.557)
Bonanno et al., 2018 [50] (n = 104)	Surgically-resected (n = 48) Biopsy tissue (n = 66)	Limited (n = 66) Extensive (n = 38)	Clone 22C3 (Dako, Carptintera, CA, USA)	Membranous ≥ 1%	25.0% (26/104)	NE
Berghoff et al., 2016 [51] (n = 32)	Biopsy tissue	Extensive	Clone 5H1 (Lieping Chen Laboratory, Yale University, USA) (Dako, Glostrup, Denmark)	Membranous ≥ 5%	34.4 (11/32)	≥5% PD-L1/TPS mOS: 8 vs. 7 months Log rank test *p* = 0.662 HR not provided
Gadgeel et al., 2018 [52] (n = 30)	Biopsy tissue	Extensive	Clone 22C3 antibody (Dako, Carpinteria, CA, USA).	Membranous ≥ 1%	10.0% (3/30)	n/a
Kim et al. 2018 [53] (n = 120)	Biopsy tissue	Limited (n = 39) Extensive (n = 81)	MAB1561/B7-H1/PD-L1 antibody (R&D Systems, Minneapolis, MN, USA	Membranous ≥ 1%	14.2% (17/120)	≥1% PD-L1/TPS, mOS: 12.0 vs. 18.0 months, HR = 1.15 (95% CI 0.76–1.73, *p* = 0.510
Wang et al., 2018 [54] (n = 94)	Biopsy tissue	Extensive (n = 52)	Clone SP142; ZSGB-BIO, Beijing, China	Membranous and/or cytoplasmic ≥ 5%	51.1% (48/94)	NE
Ichiki et al., 2018 [55] (n = 46)	Surgically-resected	Limited (n = 12) Extensive (n = 34)	Clone E1L3N, 1:800, Cell Signalling Technology, Inc., Danvers, MA, USA)	Membranous ≥ 1%	26.1% (12/46)	Overall Survival (OS): HR = 0.98 (0.94–1.02), *p* = 0.268
Liu et al., 2018 [56] (n = 80)	Surgically-resected	Limited (n = 80)	Clone SP142 (cat. no. 07309554001; 1:100; Spring Bioscience Corporation, Pleasanton, CA, USA	Membranous ≥ 5%	65.0% (52/80)	Overall survival (OS) ≥ 5% HR = 0.49 (95% CI: 0.28–0.85), *p* = 0.0110
Chung et al. 2018 [57] (n = 107)	Biopsy tissue	Extensive	Clone 22C3 pharmDx assay (Agilent Technologies)	Membranous ≥ 1%	39.0% (42/107)	n/a
Xu et al., 2019 [58] (n = 60)	Surgically-resected	Limited (n = 20) Extensive (n = 40)	Clone 2B11D11 PD-L1/CD274 antibody (cat. no. 66248-1-Ig) (ProteinTech Group, Inc., Chicago, IL, USA)	Membranous and/or cytoplasmic ≥ 5%	61.7% (37/60)	≥5% PD-L1/TPS, mOS (22.13 vs. 22.03 months) Log rank test *p* = 0.781) HR = 2.55 (95% CI: 01.18–5.51), *p* = 0.017
Zhao et al., 2019 [59] (n = 155)	Surgically-resected	Limited (n = 52) Extensive (n = 103)	Clone 22C3, PharmDx	Membranous ≥ 1%	12.9% (20/155)	≥5% PD-L1/TPS, mOS (12 vs. 57 months, Lo g rank test *p* = 0.007) HR= 5.30 (95% CI: 1.45–19.28), *p* = 0.011
Pujol et al., 2018 [60] (n = 54)	Biopsy tissue	Extensive	SP142 PD-L1 immunohistochemistry assay (Ventana Medical Systems, Inc., Tucson, AZ, USA)	Membranous ≥ 1%	1.8% (1/54)	n/a

IHC, immunohistochemistry; QIF, quantitative immunofluorescence (QIF); SCLC, small-cell lung cancer; HR, hazard ratio; N/P, not provided in the article; KM, Kaplan–Meier; OS, overall survival; mOS, median overall survival; TPS, tumour proportional score; NE, prognostic or predictive significance not evaluated; n/a, not applicable.

## Supplementary Materials

The following are available online at https://www.mdpi.com/2073-4409/9/11/2393/s1: Figure S1. Search strategy-PubMed/Scopus; Figure S2. Assessment of the quality of methodological characteristics on included studies utilising the Newcastle–Ottawa Scale (NOS); Figure S3. Begg’s funnel plots for publication bias testing for the prevalence of PD-L1 expression in SCLC; Figure S4. Forest plot of studies reporting the detection rate of PD-L1 expression in SCLC after the leave-one-out sensitivity analysis to remove outliers; Figure S5. Forest plot of subgroup analysis of the association between the use of FDA-approved PD-L1 assays or not and prevalence of PD-L1 expression; Figure S6. Forest plot of subgroup analysis of the association between the geographical distribution of published articles and prevalence of PD-L1 expression; Figure S7. Meta-regression plot showing the association of sample size with a pooled estimate of the prevalence of PD-L1 expression; Figure S8. Meta-regression plot showing the association of quality scores of the study’s methodological characteristics with the prevalence of PD-L1 expression; Figure S9. Begg’s funnel plots for publication bias testing for the prognosis of PD-L1 expression in SCLC.

## Abbreviations

PD-L1	Programmed cell death ligand-1
PD-1	Programmed cell death-1
SCLC	Small-cell lung cancer
NSCLC	Non-small cell lung cancer
ED-SCLC	Extensive diseased staged SCLC
TILS	Tumour infiltrating lymphocytes (TILs)
IHC	Immunohistochemistry
I^2^	Heterogeneity
OS	Overall survival
HR	Hazard ratio
