# Supplementary material for: Tumour PD-L1 Expression in Small-Cell Lung Cancer: A Systematic Review and Meta-Analysis
Source: Cells. 2020 Oct 31;9(11):2393. doi: 10.3390/cells9112393 (PMC7693331; doi:10.3390/cells9112393)
Supplement: Supplementary file 1 [file cells-09-02393-s001.pdf]

Supplementary Figures

PubMed Search Strategy

Search	Query	Items found
1	small cell lung cancer[MeSH Terms]	2291
2	small cell lung cancer[Title/Abstract]	2180
3	small cell lung tumor[Title/Abstract]	58
4	small cell lung tumour[Title/Abstract]	12
5	small cell lung neoplas*[Title/Abstract]	17
6	small cell lung carcinoma*[Title/Abstract]	1594
7	small cell lung malignan*[Title/Abstract]	2
8	(small cell lung cancer[MeSH Terms] OR small cell lung cancer[Title/Abstract] OR small cell lung tumor[Title/Abstract] OR small cell lung tumour[Title/Abstract] OR small cell lung carcinoma*[Title/Abstract] OR small cell lung neoplas*[Title/Abstract] OR small cell lung malignan*[Title/Abstract])	9821
9	B7-H1 antigen*[MeSH Terms]	1071
10	CD274 protein*[MeSH Terms]	1039
11	PD-L1[Title/Abstract]	4949
12	PDL1[Title/Abstract]	638
13	B7-H1[Title/Abstract]	691
14	CD274[Title/Abstract]	462
15	(B7-H1 antigen*[MeSH Terms] OR CD274 protein*[MeSH Terms] OR PD-L1[Title/Abstract] OR PDL1[Title/Abstract] OR B7-H1[Title/Abstract] OR CD274[Title/Abstract])	11772
16	8 AND 15	1747

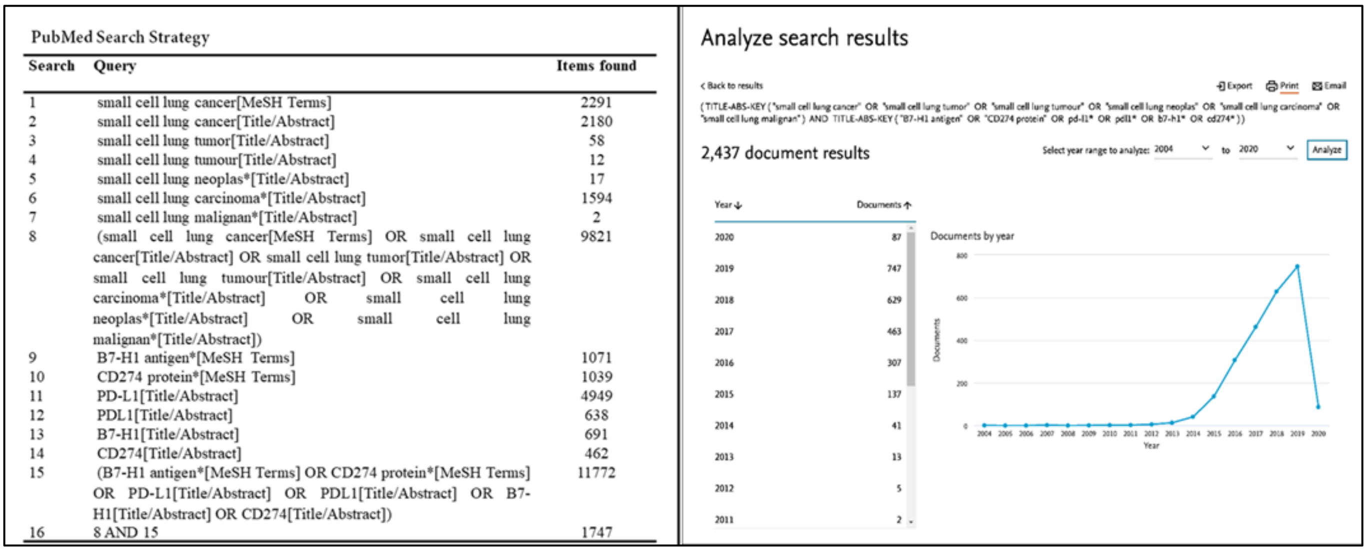

Figure S1: Search strategy -PubMed/Scopus

Author (year)	Representative sample of relevant population	Selection of the non-exposed cohort	Ascertainment of exposure	Demonstration that outcome of study does not present at the start of the study	Design of analysis controlled for cofounders	Assessment of outcome	Follow-up adequate for outcomes to occur	Quality Score
Ishii et al, 2015	*	*	*	**				5
Komiya and Madan, 2015		*	*	**				4
Schultheis et al., 2015		*	*	**		*		6
Yu et al., 2016	*	*	*	**	*	*	*	8
Takada et al., 2016		*	*	**		*		5
Antonia et al 2016	**	*	**	**	**	**	*	12
Berghoff et al., 2016	*		*	**		**	*	7
Miao et al., 2017		*	*	**		*	*	6
Chang et al., 2017	*		*	**		**	*	7
Inamura et al., 2017		*	*	**		*	*	6
Tsuruoka et al., 2017		*	*	**	*	**	*	8
Ott et al, 2017	*	*	**	**	**	**	**	12
Yasuda et al, 2018		*		**		*		4
Bonanno et al., 2018	*	*	*	**	*	*		7
Wang et al., 2018		*	*	**	*	*	*	6
Ichiki et al., 2018		*	*	**		**		7
Liu et al., 2018		*	*	**	*	**	*	8
Gadgeel et al, 2018		*	**	**	**	**	**	11
Chung et al., 2018	*	*	*	**	**	*	*	8
Kim et al., 2018	*	*	*	**	**	*	*	8
Xu et al., 2019		*	*	**		**	*	7
Zhao et al., 2019	*	*	*	**		**	*	9
Carvajal-Hausdorf et al., 2019		*	*	**	*	*		6
Pujol et al., 2019	**	*	**	**	**	**	**	13
Sun et al., 2019		*	*	**	*	**	*	8
Pas-Ares et al.,2019	**	*	**	**	**	**	**	13
Reck et al., 2020	*	*	**	**	**	**	**	12

Figure S2: Assessment of the quality of methodological characteristics on included studies utilising the Newcastle-Ottawa Scale (NOS) scale **=criterion is satisfied; *=criterion is partly satisfied; no * =criterion is not satisfied or cannot be determined. Maximum quality score=16; 0–5 points were considered low quality, 5–9 points were considered medium quality and 10–16 points were considered as high quality.

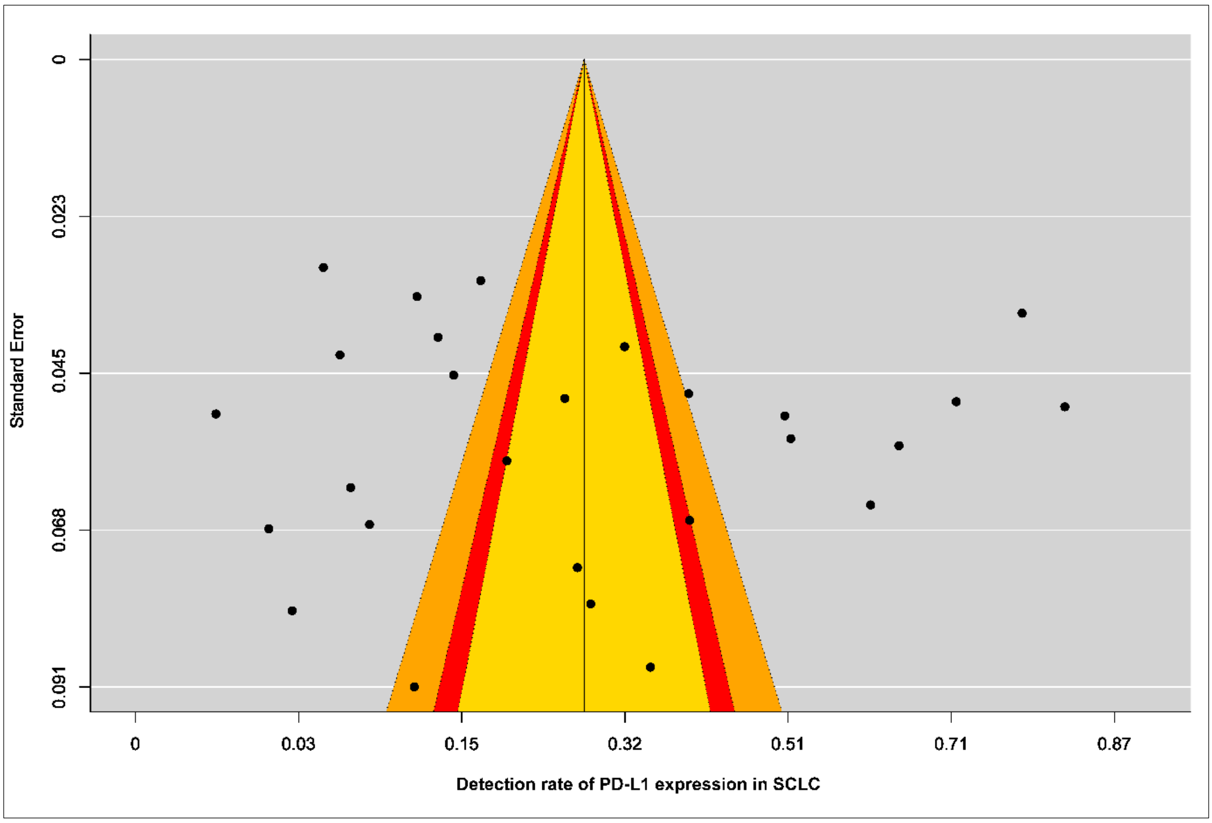

Figure S3: Begg's funnel plots with 90% CI (gold), 95% CI (red) and 99% CI (orange) for publication bias testing for the prevalence of PD-L1 expression in SCLC.

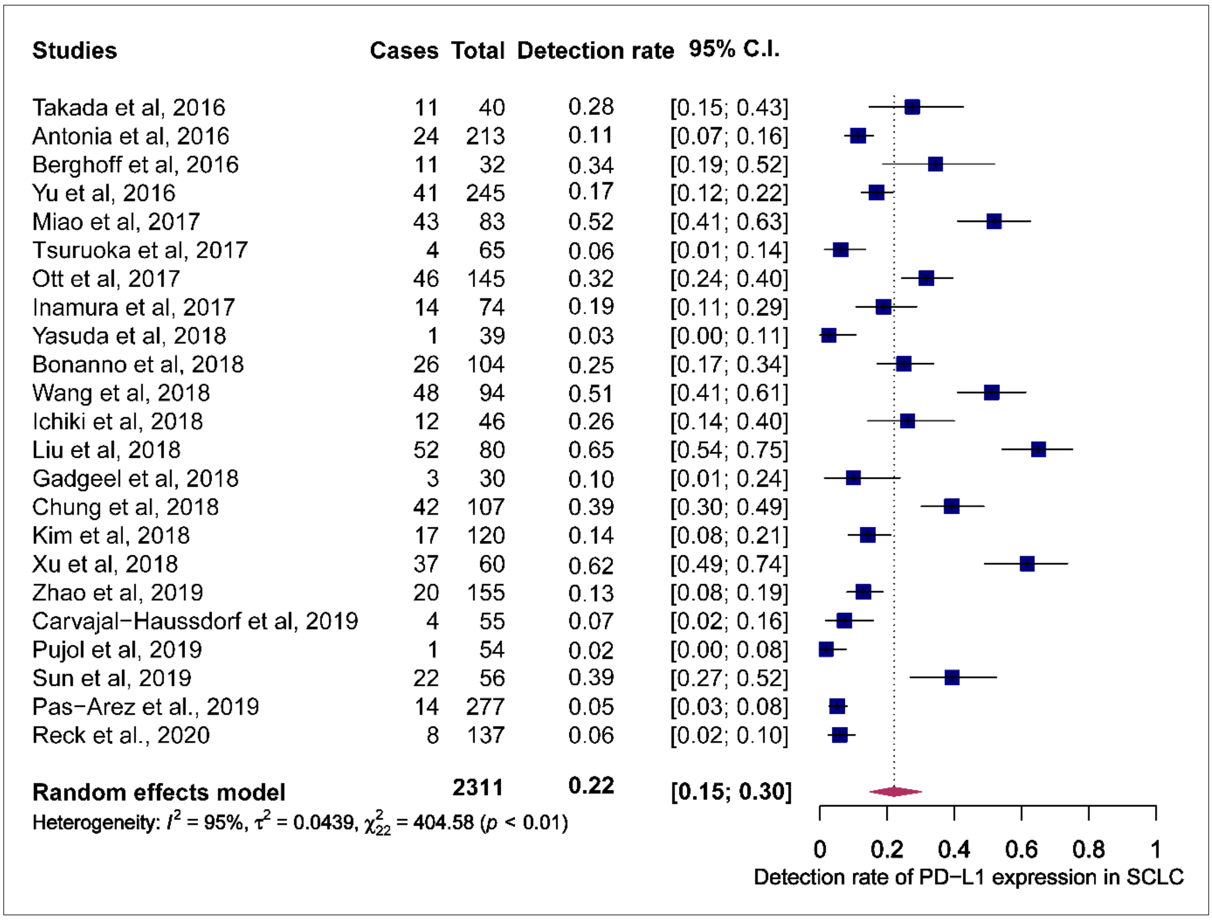

Figure S4: Forest plot of studies reporting the detection rate of PD-L1 expression in SCLC after the leave-one-out sensitivity analysis to remove outliers. The PD-L1 detection rates and 95%CI of each study is represented with a horizontal line and the square area mirrors the weight of the study A random-effect model was utilised.

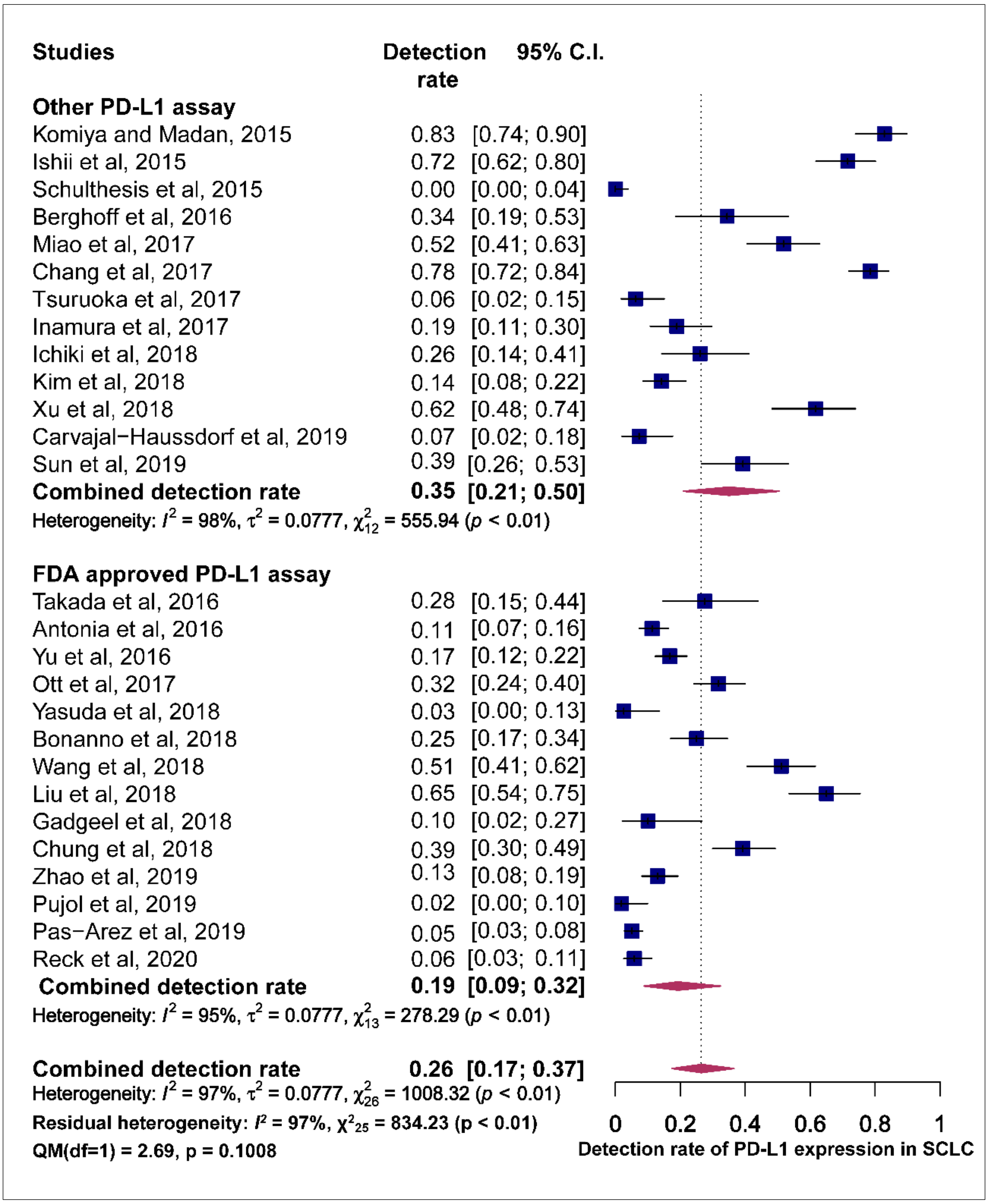

Figure S5: Forest plot of subgroup analysis of association between the use of FDA approved PD-L1 assays or not and prevalence of PD-L1 expression. The PD-L1 detection rates and 95%CI of each study is represented with a horizontal line and the square area mirrors the size effect of each study A random-effect model was utilised.

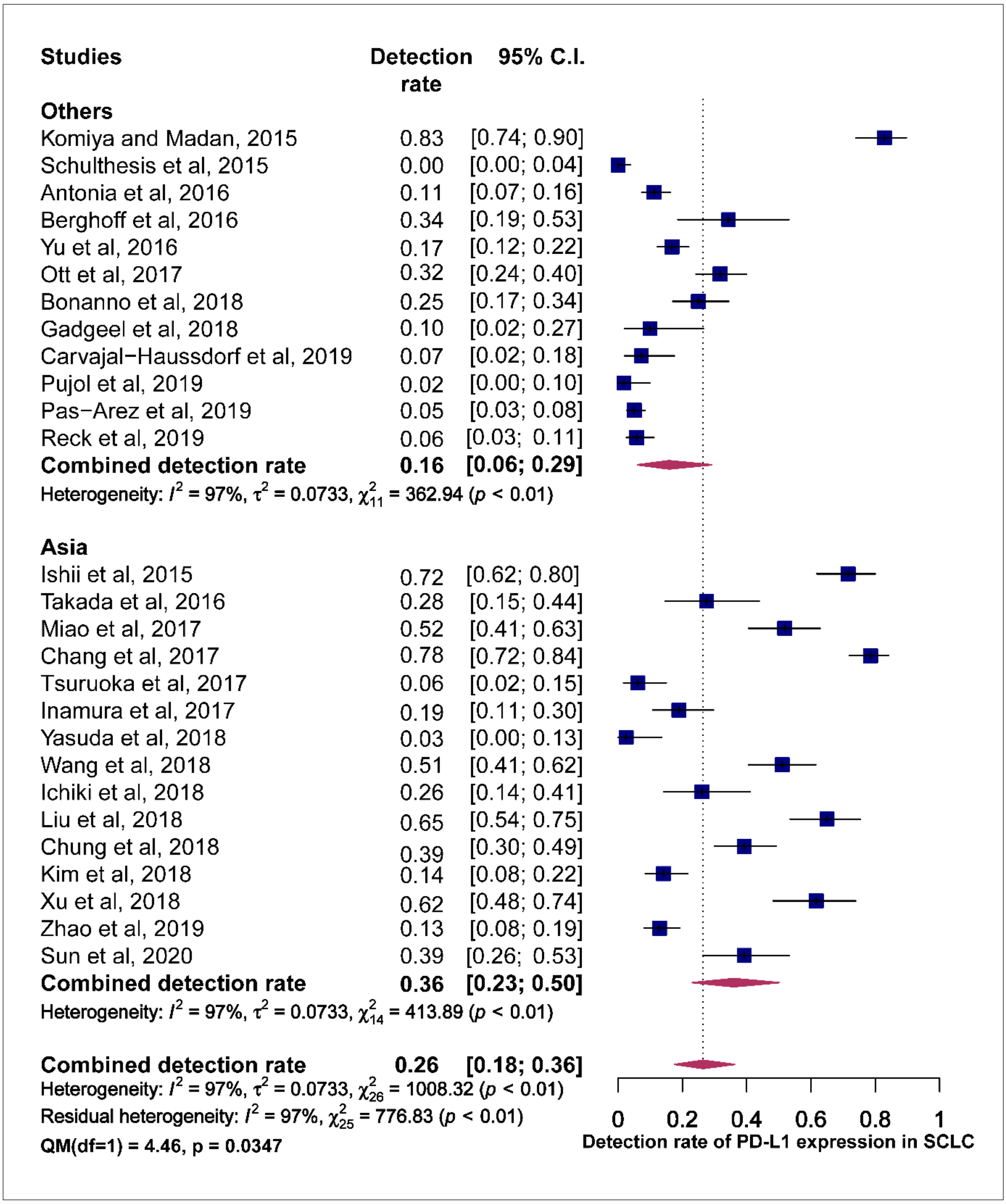

Figure S6: Forest plot of subgroup analysis of association f between geographical distribution of published articles and prevalence of PD-L1 expression. The PD-L1 detection rates and 95%CI of each study is represented with a horizontal line and the square area mirrors the weight of the study A random-effect model was utilised.

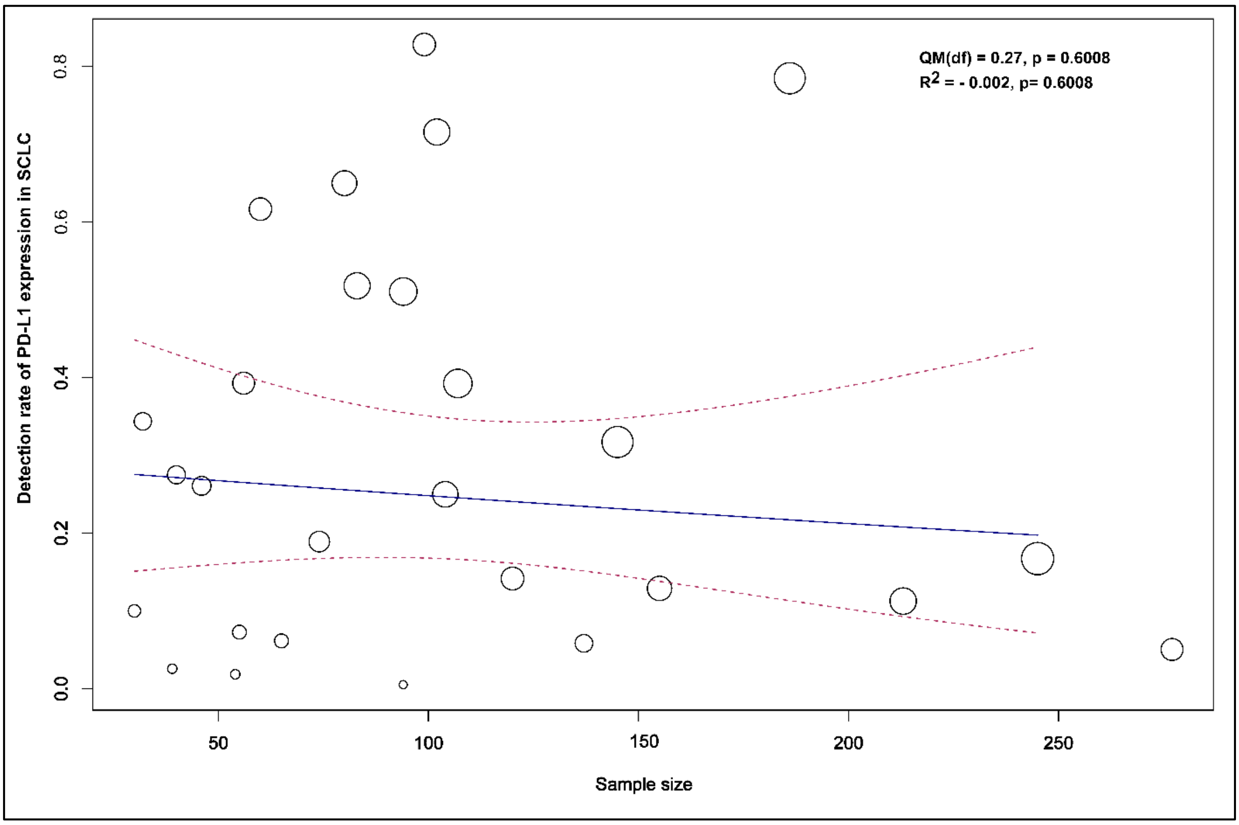

Figure S7: Meta-regression plot showing the association of sample size with pooled estimate of the prevalence of PD-L1 expression. Blue line represents the regression line of best fit, the red lines represent the lower and upper 95% confidence intervals (Cis), Each open circle represents a study. The size of the circle indicates the precision of the effect estimate and the weight given to that study

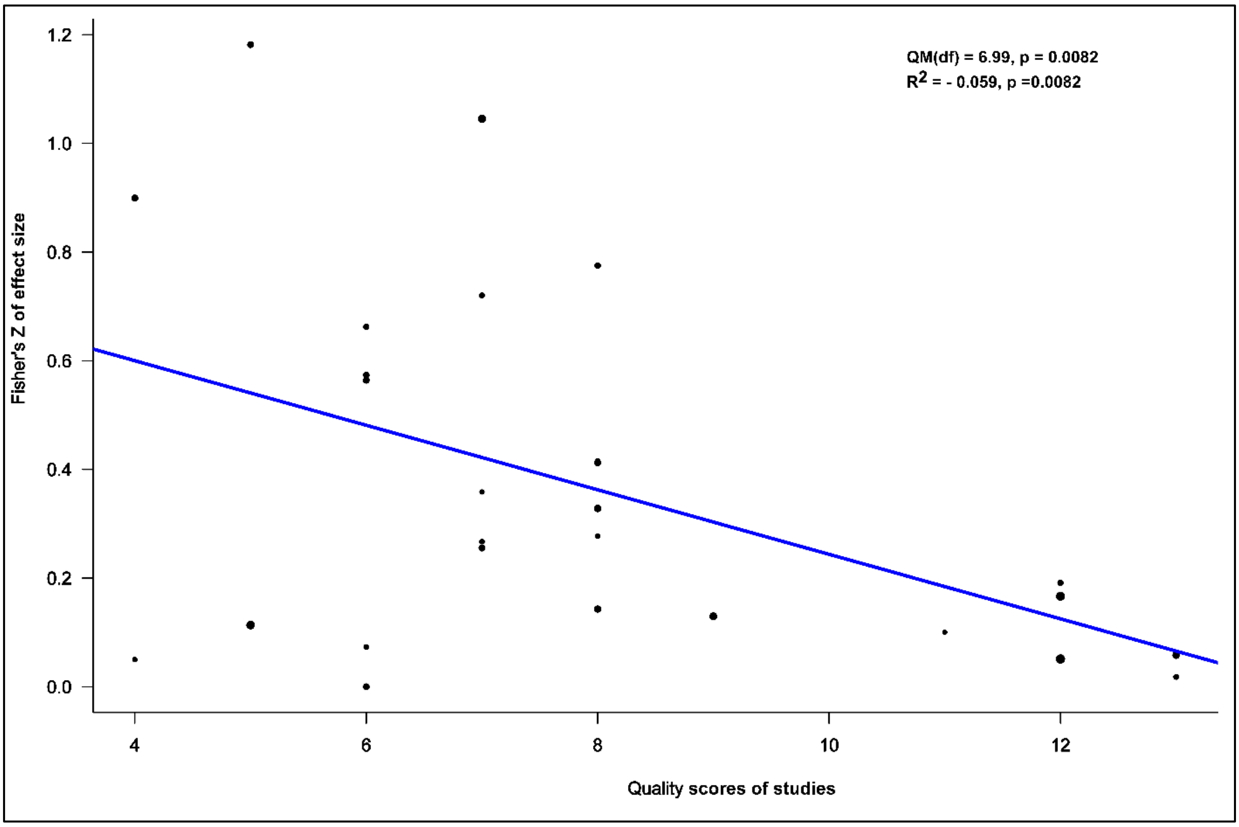

Figure S8: Meta-regression plot showing the association of quality scores of the study's methodological characteristics with pooled estimate of the prevalence of PD-L1 expression.

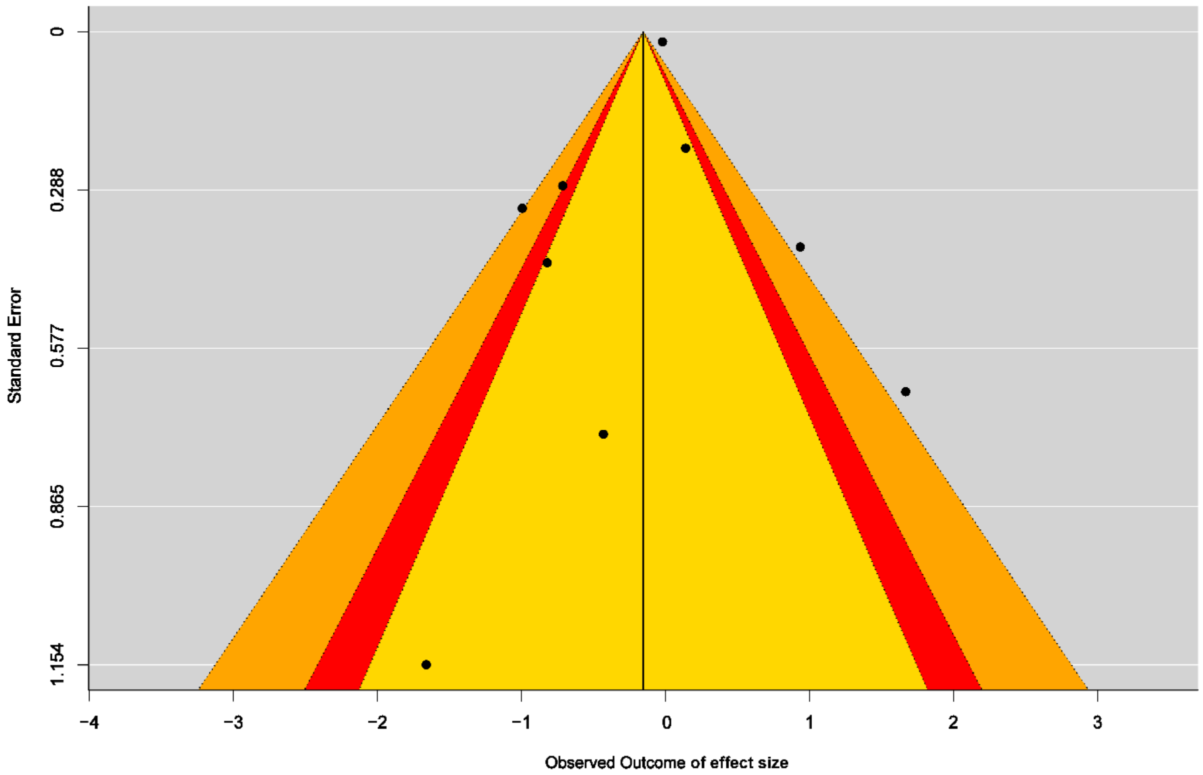

Figure S9: Begg's funnel plots with 90% CI (gold), 95% CI (red) and 99% CI (orange) for publication bias testing for the prognosis of PD-L1 expression in SCLC
